# Thyroid Dysfunction in Lung Cancer Patients Treated with Immune Checkpoint Inhibitors (ICIs): Outcomes in a Multiethnic Urban Cohort

**DOI:** 10.3390/cancers13061464

**Published:** 2021-03-23

**Authors:** Angelica D’Aiello, Juan Lin, Rasim Gucalp, Vafa Tabatabaie, Haiying Cheng, Noah A. Bloomgarden, Yaron Tomer, Balazs Halmos

**Affiliations:** 1 Department of Medicine, Montefiore Medical Center/Albert Einstein College of Medicine, Bronx, NY 10467, USA; adaiell@montefiore.org; 2 Department of Epidemiology and Population Health, Albert Einstein College of Medicine, Bronx, NY 10461, USA; juan.lin@einsteinmed.org; 3 Department of Oncology, Montefiore Medical Center/Albert Einstein College of Medicine, Bronx, NY 10461, USA; rgucalp@montefiore.org (R.G.); hcheng@montefiore.org (H.C.); 4 Division of Endocrinology, Department of Medicine, Montefiore Medical Center/Albert Einstein College of Medicine, Bronx, NY 10467, USA; vtabatab@montefiore.org (V.T.); nbloomga@montefiore.org (N.A.B.); ytomer@montefiore.org (Y.T.)

**Keywords:** immune-related adverse events, checkpoint inhibitors, immunotherapy, thyroid, NSCLC, SCLC

## Abstract

**Simple Summary:**

Which factors predispose individuals to developing immune-related adverse events (irAEs) remains unclear. In addition, the relationship between irAEs and survival outcomes warrants further investigation. We sought to investigate the association between immunotherapy-related thyroid dysfunction and demographic and clinical characteristics in a diverse urban cohort of patients with lung cancer receiving immune checkpoint inhibitors (ICIs). This study was conducted with the aim of helping to identify patients at a higher risk of experiencing irAEs and clarify whether irAEs portend a survival advantage.

**Abstract:**

We sought to characterize thyroid dysfunction and its association with baseline clinical and demographic characteristics, as well as progression-free survival (PFS), in a multiethnic cohort of lung cancer patients treated with ICIs. A retrospective chart review of lung cancer patients receiving an anti-PD1 or PD-L1 agent was performed. Multivariate Cox proportional hazards were fitted to compare time to thyroid dysfunction among race subgroups controlling for age, gender, treatment type, and duration. Thyroid dysfunction was based on laboratory testing; clinical symptoms were not required. PFS at a 24-week landmark analysis point among patients with and without thyroid dysfunction was compared using a log-rank test. We identified 205 subjects that received ICIs, including 76 (37.1%) who developed thyroid dysfunction. Rates of thyroid dysfunction by one year occurred at similar frequencies among all races (*p* = 0.92). Gender and concurrent chemotherapy showed no significant association with thyroid dysfunction (*p* = 0.81 and *p* = 0.67, respectively). Thyrotoxicosis occurred at higher rates in Black (25, 31.6%) subjects than in White (7, 16.7%) and Hispanic (8, 12.7%) subjects when employing the log-rank test (*p* = 0.016) and multivariate Cox regression (HR 0.48, *p* = 0.09 for White and HR 0.36, *p* = 0.01 for Hispanic compared to Black subjects). PFS was similar among subjects with and without thyroid dysfunction when applying the log-rank test (*p* = 0.353). Gender, concurrent treatment with chemotherapy, and PFS were not associated with thyroid dysfunction in patients receiving ICIs; however, Black race was a risk factor for thyrotoxicosis. The mechanisms underlying the role of race in the development of irAEs warrant further study.

## 1. Introduction

Immune checkpoint inhibitors (ICIs) are now being broadly utilized as effective therapies for multiple malignancies, including lung cancer [1,2]. ICIs currently in use involve two key immune regulatory pathways—the programmed cell death protein 1 (PD-1), and its ligand, PD-L1, as well as anti-CTLA-4 [3]. PD-1 is a transmembrane protein expressed on immune cells that binds to PD-L1 present on many tissues, including tumor cells. The PD-1:PD-L1 interaction has multiple effects, including inhibition of the cytotoxic T-cell function and tumor cell apoptosis. Monoclonal antibodies targeting PD-1 (nivolumab and pembrolizumab) and PD-L1 (durvalumab, atezolizumab, and avelumab) have been engineered to interrupt these checkpoints, thereby enhancing immune-mediated antitumor responses.

Through their unique activation of T-cells, ICIs have novel toxicity profiles compared to traditional chemotherapy. These side effects are collectively known as immune-related adverse events (irAEs). While irAEs can affect any organ tissue, they most commonly involve endocrine, skin, gastrointestinal, pulmonary, and musculoskeletal systems [4]. The clinical severity of irAEs ranges from asymptomatic or mild manifestations to more severe toxicities, such as colitis, pneumonitis, and Steven–Johnson Syndrome [5].

The toxicities associated with inhibitors of CTLA-4 and PD-1 are distinct, and several mechanisms have been proposed to explain these differences. Both CTLA-4 and PD-1 checkpoints are involved in maintaining self-tolerance. CTLA-4 attenuates T-cell activation at an early step in the immune response, while PD-1 inhibits T cells at later stages within peripheral tissues [6]. CTLA-4 regulates T-cell responses by (1) directly competing with CD28 for the B7 ligand on antigen-presenting cells (APCs) in one of the earliest negative regulatory steps [7], and (2) its expression on T-regulatory (Treg) cells that is required for the Treg function in inhibiting autoreactive T-cells [8]. PD-1 expressed during T-cell activation engages its ligand PD-L1, which also serves to counter the positive signaling events triggered by the T-cell receptor (TCR) and peptide–major histocompatibility complex (MHC) interaction. The PD-1 pathway ultimately limits autoimmunity, as demonstrated by prior research. For example, mice genetically deficient in the gene encoding PD-1 were found to develop accelerated autoimmunity, including myocarditis, lupus-like disease, diabetes, and pneumonitis [9,10,11,12].

There are several mechanisms by which irAEs are thought to develop, including triggering autoimmunity in genetically susceptible individuals by upregulating autoreactive T-cells that escaped the central and peripheral tolerance mechanisms, cross-reactivity of neoantigens released from cancer cells and normal tissue, and cytokine production. In non-small cell lung cancer (NSCLC) patients treated with pembrolizumab, preexisting anti-thyroid antibodies (a biomarker of the genetic susceptibility to autoimmune thyroiditis) were observed in a subset of those that developed thyroid dysfunction, suggesting that irAEs may result from the enhancement of an underlying susceptibility to autoimmunity [13]. In one report of ICIs-related myocarditis, similar T-cell populations were observed in both the tumor and myocardium, indicating that the T-cell clones may have been reactive against an antigen shared between the tumor and myocardial cells [14]. More evidence to support cross-reactivity between antigens in tumor and normal tissue as a mechanism of irAEs comes from the observation that vitiligo commonly occurs in patients receiving ICIs for the treatment of melanoma [15]. Finally, cytokines may play a role in the development of irAEs. For example, in one study of patients treated with the CTLA-4 inhibitor ipilimumab that developed colitis, elevated levels of interleukin-17 were also observed [16].

Elucidating which factors predispose and protect individuals from the development of irAEs is an area of active study. Prior research has explored both the influence of gender and concurrent chemotherapy as risk factors for irAEs. While one study of patients with metastatic melanoma and NSCLC treated with PD-1 inhibitors found that women were more likely to experience irAEs compared to men, another study showed a female sex to be protective [17,18]. Concurrent chemotherapy has been associated with lower rates of irAEs based on data from prior randomized controlled trials (RCTs)—a finding consistent with the anticipated immunosuppressive effect of chemotherapy [1,2,19]. The contribution of race to the development of irAEs is unclear and there is a paucity of literature investigating this issue. 

Endocrine perturbations are among the most commonly encountered irAEs. While hypophysitis occurs more frequently with anti-CTLA-4 therapy, thyroid dysfunction is more common with PD-1 and PD-L1 inhibitors [20,21,22]. The incidence of ICIs-related thyroid dysfunction is unclear, with reported rates ranging from 3 to 20% [23,24]. ICIs-related thyroid dysfunction encompasses both thyrotoxicosis and hypothyroidism, which often present sequentially in a thyroiditis-like illness. Clinical manifestations include weight loss, heat intolerance, and palpitations in the case of thyroid hormone excess, as well as weight gain, hair loss, and constipation associated with hypothyroidism.

We sought to explore demographic and clinical risk factors for irAEs in patients receiving anti-PD1 or anti-PDL1 in a large multiethnic cohort of patients with lung cancer treated with ICIs, allowing for particularly robust comparisons of ICIs alone versus concurrent chemotherapy, as well as racial subgroups. We focused our analyses on ICIs-related thyroid dysfunction, as it is one of the most common irAEs. Finally, we chose PFS in a landmark analysis of 24 weeks in order to minimize the risk of survivorship bias [25].

## 2. Materials and Methods

### 2.1. Subjects

The project was approved by the Einstein-Montefiore Institutional Review Board (IRB number: 2013-2570). The medical records of lung cancer patients treated with ICIs at Montefiore Medical Center from January 2016 to July 2019 were retrospectively reviewed. Subjects included in the analysis had documentation of a normal baseline thyroid function within one year prior to starting ICIs. Patients who were prescribed either levothyroxine or methimazole within one year prior to starting ICIs, regardless of baseline thyroid-stimulating hormone (TSH), were excluded. Standard dosing of ICIs was used according to guidelines. Dosing was not altered as a result of endocrine irAEs that were asymptomatic or mild, in accordance with current guidelines [5]. 

### 2.2. Definitions

Thyrotoxicosis and hypothyroidism were defined as a thyroid-stimulating hormone (TSH) level less than 0.4 and greater than 4.5 uU/mL, respectively, according to institutional laboratory reference ranges. The time to thyroid dysfunction was defined as the time from ICIs initiation to the first documented TSH level consistent with either thyrotoxicosis or hypothyroidism. For our definition of thyrotoxicosis or hypothyroidism, clinical symptoms were not required. While the terms thyrotoxicosis and hyperthyroidism are often used interchangeably, in this manuscript, the term thyrotoxicosis was chosen as this has classically been used to describe a thyroiditis-type illness, which is the most common presentation of thyroid irAEs.

### 2.3. Outcomes

The primary outcome was the rate of thyroid dysfunction based on serial TSH monitoring occurring over 365 days from ICIs initiation. The primary outcome was compared among race, gender, and treatment subgroups. The secondary outcome was progression-free survival (PFS). PFS was compared among thyroid dysfunction subgroups for subjects with advanced NSCLC defined by investigator-determined Response Evaluation Criteria in Solid Tumors (RECIST) version 1.1. Due to the risk of survivorship bias [25], we conducted a 24-week landmark analysis of PFS including only patients who were alive at 6 months after starting ICIs. Rates of other irAEs were also collected. 

### 2.4. Statistical Analyses

Univariate associations comparing characteristics between subjects with and without thyroid dysfunction (hypothyroidism or thyrotoxicosis) were performed using t-tests or the Wilcoxon ranked sum test for continuous variables and chi-square test or Fisher’s exact test for categorical variables, as indicated in Results. Multivariate Cox proportional hazards models were fitted to compare the time to thyroid dysfunction among race groups controlling for age, gender, treatment type, and duration. The hazard ratio and 95% confidence interval of each factor involved in multivariate cox model analysis were provided. Kaplan–Meier survival curves were plotted to visualize and log-rank tests were performed to test the difference in time to thyroid dysfunction by race, gender, and treatment groups. Descriptive statistics were used to report the time to thyroid dysfunction and other irAE data. Progression-free survival (PFS) among thyroid dysfunction subgroups was visualized and compared using the Kaplan–Meier plot and log-rank test. 

*p*-values less than 0.05 were considered statistically significant. Analyses were conducted using SAS 9.4 (SAS Institute, Cary, NC, USA). Statistical significance was determined by a two-sided *p*-value < 0.05.

## 3. Results

### 3.1. Study Population

A total of 316 patients with lung cancer were treated with ICIs during the study period. A total of 111 patients were excluded: Sixty-one patients were excluded due to a lack of either baseline TSH (*n* = 28) or repeat TSH measurements while on ICIs treatment (*n* = 33), and another 50 patients were excluded due to abnormal baseline TSH (*n* = 42) or treatment with levothyroxine or methimazole within one year prior to starting ICIs (*n* = 8). 

Two-hundred and five subjects were included in the final analysis. The baseline characteristics of the study population are shown in Table 1. The study group included 187 (91.2%) subjects with NSCLC and 18 (8.8%) subjects with small cell lung cancer (SCLC), of which 174 (84.9%), 30 (14.6%), and 1 (0.5%) had stage IV, III, and II disease, respectively. The median age at treatment initiation was 66 years (33–87). Gender was represented equally, and the race distribution was 38.5% Black, 30.7% Hispanic, 20.5% White, 1.9% Asian, and 9.3% “other” or unknown. The majority of subjects were treated with immunotherapy alone (142, 69.3%) versus combination with chemotherapy (63, 30.7%). The median duration of treatment overall was 15.0 weeks (0.1–145.0) and was similar among both immunotherapy and combination chemotherapy subgroups (15.2 and 15.1, respectively).

### 3.2. Evaluation of Thyroid Dysfunction

Of the 205 subjects studied, 76 (37.1%) developed thyroid dysfunction. Of the 76 patients with thyroid dysfunction, 9 (11.8%) were Stage III and 67 (88.2%) were Stage IV. There were 34 subjects (16.6%) who developed hypothyroidism only, 24 (11.7%) who developed thyrotoxicosis that subsequently became euthyroid, 14 (6.8%) who developed thyrotoxicosis followed by hypothyroidism, and 4 (2.9%) who developed thyrotoxicosis and remained thyrotoxic. Of note, those subjects with thyrotoxicosis not followed by euthyroid or hypothyroid states all had fewer than two follow-up TSH values after the onset of thyrotoxicosis.

Univariate associations comparing characteristics between subjects with and without thyroid dysfunction are depicted in Table 2. There was no association between age, gender, race, BMI (body mass index), or treatment type with thyroid dysfunction overall (hypothyroidism or thyrotoxicosis) or with hypothyroidism alone; however, thyrotoxicosis was only significantly associated with race (*p* = 0.034). As expected, both thyroid dysfunction overall and hypothyroidism were associated with a longer treatment duration (*p* = 0.002 and *p* = 0.001, respectively), and thyrotoxicosis was more frequent with a longer treatment duration, but this did not reach statistical significance (*p* = 0.067).

**Figure 1:**
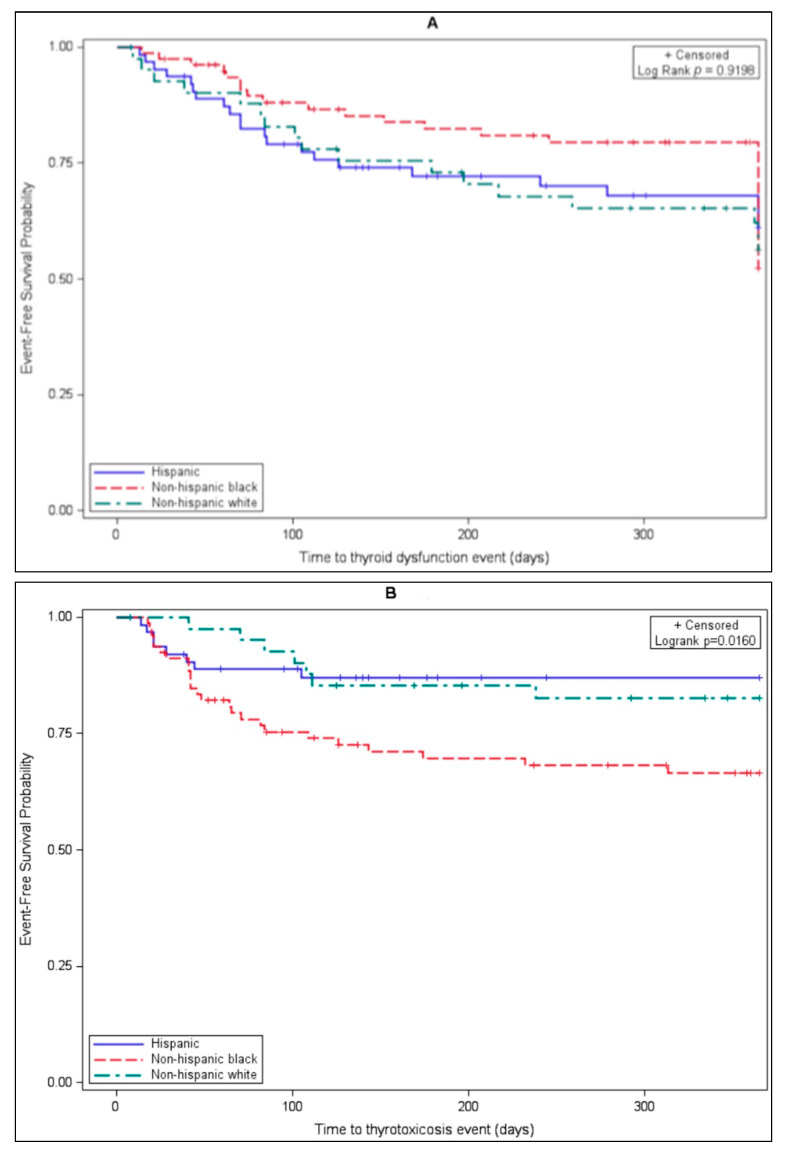

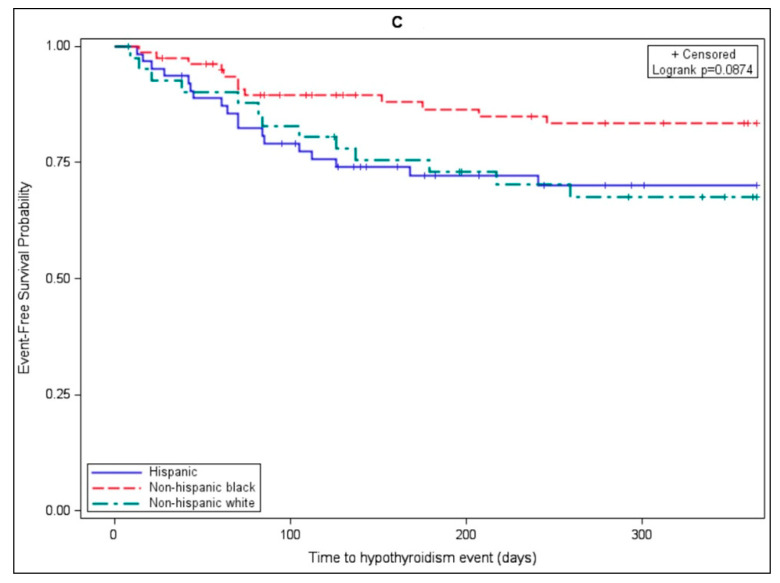
Time to (**A**) thyroid dysfunction, (**B**) thyrotoxicosis, and (**C**) hypothyroidism.

Rates of thyroid dysfunction (thyrotoxicosis or hypothyroidism) by one year occurred at similar frequencies among all major races (Figure 1A, *p* = 0.92). At one year, rates of thyrotoxicosis were significantly higher in Black (25/79, 31.6%) subjects than in White (7/42, 16.7%) and Hispanic (8/63, 12.7%) subjects (Figure 1B, *p* = 0.016). In contrast, hypothyroidism occurred more often at one year in White (13/42, 31.0%) and Hispanic (18/63, 28.6%) subjects than in Black (12/79, 15.2%) subjects, though this difference did not reach statistical significance (Figure 1C, *p* = 0.090).

Multivariate Cox regression analyses comparing thyroid dysfunction among patient subgroups controlling for age, gender, treatment type, and treatment duration were performed (Table 3). Hypothyroidism was more likely to occur in Hispanic (HR 2.466, *p* = 0.0191) and White patients (HR 2.182, *p* = 0.0535) compared to Non-Hispanic Black subjects. Thyrotoxicosis was most likely to occur in Black subjects compared to Hispanic subjects (HR 0.360, *p* = 0.0129); this trend was also observed compared to White and Asian/other subgroups, though it did not reach statistical significance (*p* = 0.09 and 0.08, respectively).

For those subjects with thyrotoxicosis (*n* = 42), the median onset was 6.0 weeks (2.49–44.8). For those subjects with hypothyroidism only (*n* = 34), the median onset was 10.0 weeks (1.3–37.0). Of subjects with thyrotoxicosis followed by hypothyroidism (*n* = 14), the median time to thyrotoxicosis and hypothyroidism was 4.0 and 7.2 weeks, respectively. Of subjects with thyrotoxicosis who subsequently became euthyroid, but not hypothyroid, the median time to a euthyroid state was 4.0 weeks.

Of the 205 subjects included in the analysis, only 14 underwent thyroid antibody testing; for the majority of these individuals (13/14), antibody testing occurred after starting ICIs. Among those 14 subjects who underwent antibody testing, 5 had positive antibodies: Two with both anti-thyroid peroxidase (anti-TPO) and thyroid-stimulating immunoglobulin (TSI), and 3 with anti-TPO alone. These included 3 White subjects, 1 Hispanic subject, and 1 Black subject. All subjects with positive antibodies experienced thyrotoxicosis followed by hypothyroidism.

### 3.3. Clinical Presentation

Symptoms associated with thyrotoxicosis in our cohort included tremors, anxiety, palpitations, and insomnia; however, only 3 subjects with thyrotoxicosis reported such symptoms. Among subjects with thyrotoxicosis, only one required treatment with methimazole, later becoming hypothyroid and receiving treatment with levothyroxine. With the exception of 4 subjects, all other subjects with thyrotoxicosis later became either euthyroid or hypothyroid. Those subjects with thyrotoxicosis not followed by euthyroid or hypothyroid states all had fewer than two follow-up TSH values after the onset of thyrotoxicosis due to death or the end of the study period. Subjects with hypothyroidism were largely asymptomatic; only 4 subjects reported symptoms attributable to hypothyroidism (fatigue and constipation). Of subjects with hypothyroidism (*n* = 48), 40.0% (*n* = 19, 9.2% of the total cohort) received treatment with levothyroxine. Furthermore, thyrotoxicosis preceded hypothyroidism in 68.4% (*n* = 13) of those who received levothyroxine. Among subjects with thyroid dysfunction, 17.1% (*n* = 13, 6.3% of the total cohort) were referred to endocrinology for evaluation and management.

Other irAEs were observed in 11.2% (*n* = 23) of subjects, including pneumonitis (10, 4.9%), dermatitis (8, 3.9%), colitis (2, 1.0%), inflammatory arthritis (2, 1.0%), and nephritis (1, 0.5%). The majority of irAEs occurred in subjects receiving ICIs alone (16, 69.6%) versus concurrent chemotherapy (7, 30.4%). Of subjects with thyroid dysfunction (*n* = 76), other irAEs co-occurred in 11.8% (*n* = 9). Of the 23 subjects with other irAEs, 30.4% (*n* = 7) had thyroid dysfunction.

### 3.4. Association of Thyroid Dysfunction and Survival Outcomes

**Figure 2:**
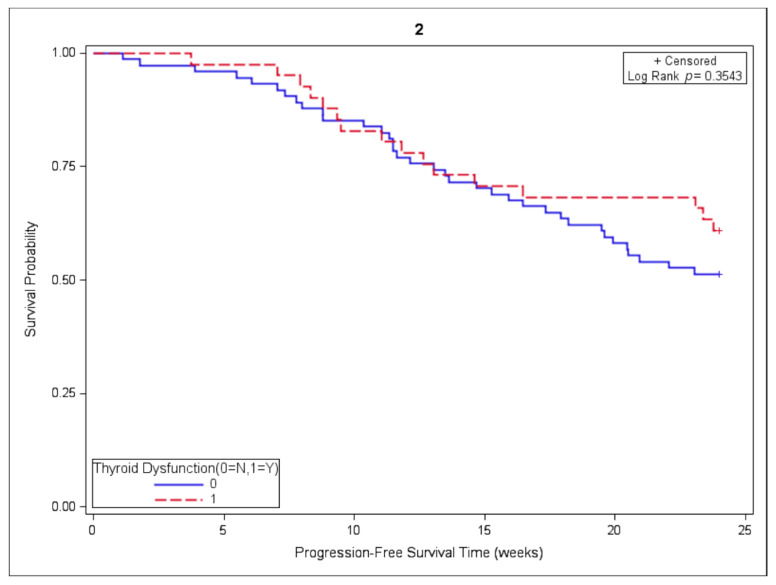
Association of thyroid dysfunction and progression-free survival (PFS).

Of the initial cohort, 116 subjects were included in the 24-week landmark PFS analysis, including 42 subjects with and 74 without thyroid dysfunction. The median PFS was not reached in either group. PFS was similar among subjects with and without thyroid dysfunction when employing the chi-square test (*p* = 0.3206) and log-rank test (*p* = 0.3543), as depicted in Figure 2.

## 4. Discussion

Thyroid dysfunction and its association with baseline clinical and demographic characteristics, as well as PFS, were studied in a multiethnic cohort of lung cancer patients treated with ICIs. Chemotherapy (despite its immunosuppressive effect) and gender (despite its association with autoimmune thyroid diseases) were not associated with thyroid dysfunction during ICIs therapy. Similarly, treatment outcomes were also not associated with thyroid dysfunction, despite previously reported associations [13,26,27]. Intriguingly, race and ethnicity were associated with thyroid dysfunction, with a Black race being associated with thyrotoxicosis and White or Hispanic race/ethnicity being associated with hypothyroidism. To the best of our knowledge, this is the first study to demonstrate the influence of race/ethnicity on the development of ICIs-related thyroid dysfunction.

### 4.1. Biology and Epidemiology

The etiology of non-ICIs-related autoimmune thyroid diseases (AITD) has been shown to be influenced by genetic factors, sex hormones, gender, and race. The major genes associated with AITD include HLA-DR, CTLA-4, PTPN22, CD40, the TSH receptor, and thyroglobulin genes [28]. In addition, sex hormones are known to influence the immune function and this may partly explain the higher incidence of autoimmune disease in females compared to males, even though genetic factors on the X-chromosome may also play a role [29,30,31,32]. Prior research has suggested that AITD occur differentially based on race. While hypothyroidism occurred most often in Whites, thyrotoxicosis, including Graves’ disease, occurred more often in Blacks, even when controlling for risk factors such as smoking [33,34,35]. Though a Black race has been associated with lower baseline TSH reference ranges [36], a higher prevalence of thyrotoxicosis was still observed when using a TSH cut-off as low as 0.1 [35]. Though several studies have assessed the association of HLA-DR alleles with Graves’ disease in Black patients, the results have been variable and more studies examining other susceptibility alleles are needed [37,38,39,40,41,42]. How genetics, hormones, gender, race, and environmental exposures relate to ICIs-related thyroid disease is not known.

The pathogenesis of ICIs-related thyroid dysfunction and the relationship with non-ICIs-related autoimmune thyroid disease is an area of active research. Subjects treated with ICIs have been reported to have elevated thyroid antibodies, including anti-TPO, anti-thyroglobulin, and thyrotropin (TSH) receptor antibodies [13,43,44]. In addition, ICIs-related Graves’ disease has been reported in patients treated with the CTLA-4 blocker ipilimumab [45]. While the majority of subjects in our study did not undergo any antibody testing, roughly one-third of those that did exhibited antithyroid antibodies, suggesting that in some cases of ICIs-induced thyroiditis, the etiology is autoimmune. Another study proposed a phenotype distinct from non-ICIs-related autoimmune disease—one governed by circulating CD56, CD16, and natural killer (NK) cells, rather than antibodies. However, these data need replication [46]. Whether or not ICIs-related thyroid dysfunction shares the same mechanisms as non-ICIs-related autoimmune thyroid disease warrants further study.

### 4.2. Clinical Impact

This study found the incidence of thyroid dysfunction in patients receiving ICIs for lung cancer to be 37.1%, which is a rate higher than previously reported; however, the prevalence of ICIs-related thyroid dysfunction is difficult to estimate given inconsistent definitions of thyroid dysfunction and potential selection bias [47]. Existing data from RCTs have shown rates of thyroid dysfunction ranging from 3 to 20% [23,24]. It is possible that RCTs tend to report primarily clinically significant thyroid dysfunction, i.e., symptomatic or requiring treatment. While our study did not investigate rates of “symptoms” among subjects with thyroid dysfunction, it did show that only 9.3% (*n* = 19) of subjects required treatment, suggesting that most thyroid dysfunction is asymptomatic or “subclinical”, and may not require treatment. Furthermore, the criteria we used to define thyroid dysfunction based on TSH alone may also overestimate the prevalence of thyroid dysfunction by including “subclinical” cases.

Given the immunosuppressive effect of chemotherapy, we postulated that irAEs might be less frequent among patients receiving ICIs with concurrent chemotherapy compared to ICIs alone, consistent with prior RCTs [1,2,19]. Though the thyroid dysfunction frequency was not lower in patients receiving concurrent chemotherapy, other irAEs did occur less often in patients receiving ICIs with concurrent chemotherapy. Of note, the median duration of treatment was similar among patients receiving ICIs alone versus ICIs and concurrent chemotherapy, suggesting that survivorship bias did not contribute to this differential development of irAEs. While prior research has shown that the risk of thyrotoxicosis is greater with PD-1 inhibitors than with PD-L1 inhibitors, this finding was not replicated in our study [24].

In addition, our study showed no effect of gender on rates of thyroid dysfunction. This finding is somewhat unexpected given the higher incidences of non-ICIs-related autoimmune disease in women and may suggest that ICIs-induced thyroiditis has a different etiology than autoimmune thyroiditis.

Our study also demonstrated that Black subjects had significantly higher rates of thyrotoxicosis and lower rates of hypothyroidism, though the latter did not reach statistical significance. While more Black subjects experienced thyrotoxicosis than other racial subgroups, this was largely transient and did not require treatment, with the exception of one subject, who received methimazole. The mechanisms underlying this association are unknown, but the fact that previous studies have shown an association between a Black race and non-ICIs-related thyrotoxicosis suggests shared mechanisms.

The relationship between ICIs-related thyroid dysfunction and the treatment response has been a topic of research in a number of retrospective studies [13,26,27,48,49,50,51,52] (Table 4). Notably, none of these studies employed a landmark analysis approach. Given the potential impact of survivorship bias [25], the results of such studies must be interpreted with caution. Patients benefiting from ICIs typically receive treatment for longer intervals, increasing the exposure time and confirmation period for the development of irAEs. Using a 24-week landmark analysis, we did not observe any differences in PFS among subjects with and without thyroid dysfunction. Nonetheless, prospective randomized controlled trials are necessary to further evaluate the relationships of irAEs, the treatment response, and survival outcomes.

### 4.3. Recommendations for Monitoring and Management

This study demonstrated a high frequency of thyroid dysfunction among lung cancer subjects treated with ICIs. Though only about a third of subjects with thyroid dysfunction ultimately required treatment, those that did included about 9% of the study population, making thyroid dysfunction one the most frequent irAEs. The onset of thyroid perturbations occurred early on during therapy, around the two-month time point after treatment initiation. These findings support the need for close thyroid monitoring as part of supportive care during ICIs therapy and consistent reporting during RCTs. Given the possible role of thyroid antibodies (mostly TPO and thyroglobulin antibodies) in the development of ICIs-related thyroid dysfunction, the testing of TPO and thyroglobulin antibodies prior to ICIs initiation should be considered as positive antibodies may predict a higher likelihood of the development of thyroiditis during ICIs therapy. Furthermore, our observation that Black subjects experience higher rates of thyrotoxicosis calls for increased vigilance for these irAEs in Black patients and an increased awareness among oncologists.

### 4.4. Directions for Future Research

Minority groups have historically been under-represented in clinical research, with Black patients comprising less than 4% of all those enrolled in trials supporting the approval of ICIs for lung cancer [53]. The inclusion of minorities in clinical trials must become a priority in order to better understand how race may be associated with the response to and toxicities of immunotherapy. In addition, prospective randomized controlled trials using landmark analyses are necessary to further investigate whether irAEs have any relationship with treatment outcomes. Yet another area for future study involves the risk of endocrine, including thyroid irAEs, in patients with pre-existing autoimmune disease. Several studies have investigated the impact of immunotherapy on patients with baseline thyroid dysfunction or thyroid antibodies, presenting mixed findings [49,50,54]. While the most robust evidence regarding irAEs comes from prospective clinical trials, patients with autoimmune disease were excluded, so the safety of immunotherapy for this population ultimately remains unclear [55]. Finally, given emerging data suggesting that the discontinuation of immunotherapy versus indefinite treatment may be an appropriate option, the relationship between the treatment duration and irAEs should be further assessed given the continued risk for toxicity with prolonged treatment [56].

### 4.5. Limitations

The main limitation of our study is that it is retrospective. Though this study’s total sample size was robust compared to other similar retrospective studies, smaller subgroup sizes limit comparisons and conclusions. Larger, controlled prospective studies are necessary to conclusively answer the research questions of our study. In addition, this study’s definition of thyroid dysfunction based on the TSH level rather than symptoms or treatment may overestimate the incidence of thyrotoxicosis and hypothyroidism. Furthermore, the authors recognize that information regarding pre-existing thyroid antibodies is lacking as this type of testing was not routinely conducted prior to treatment, in accordance with current treatment guidelines [5].

## 5. Conclusions

We observed that lung cancer patients undergoing treatment with ICIs commonly experience ICIs-related thyroid dysfunction, which typically occurs early in the course of therapy, and requires intervention in close to 10% of patients. Furthermore, ICIs-related thyroid dysfunction showed associations with race, but not gender, treatment regimen, or PFS. To the best of our knowledge, this represents the largest study investigating ICIs-related thyroid dysfunction in a mixed NSCLC and SCLC population, as well as the first report demonstrating the influence of race on the development of ICIs-related thyroid dysfunction. While the pathogenesis of ICIs-related thyroid dysfunction and other irAEs is unclear, the role of race demonstrated by our study warrants further investigation. Uncovering the mechanisms underlying racial differences in ICIs-related thyrotoxicosis may lead to a better understanding of this complication of ICIs therapy and hopefully, will enable the development of treatment or prevention modalities.

## Figures and Tables

**Table 1:** Baseline characteristics.

Category	*n* = 205
Age (years) at treatment start, median (range)	66 (33–87)
Sex, *n* (%)	
Male	103 (50.2)
Female	102 (49.8)
Ethnicity, *n* (%)	
Black	79 (38.5)
Hispanic	63 (30.7)
White	42 (20.5)
Asian	2 (1.9)
Other or Unknown	19 (9.3)
Stage, *n* (%)	
II	1 (0.5)
III	30 (14.6)
IV	174 (84.9)
Lung cancer type, *n* (%)	
NSCLC	187 (91.2)
SCLC	18 (8.8)
Treatment, *n* (%)	
Immunotherapy only	142 (69.3)
Immunotherapy + chemotherapy	63 (30.7)
Immunotherapy type, *n* (%)	
Pembrolizumab	101 (49.3)
Nivolumab	65 (31.7)
Durvalumab	26 (12.7)
Atezolizumab	20 (9.8)
Duration of treatment (weeks), median (range)	
Overall	15.0 (0.1–145.0)
Immunotherapy only	15.2 (0.1–145.0)
Immunotherapy + chemotherapy	15.1 (0.1–135.0)

NSCLC, non-small cell lung cancer; SCLC, small cell lung cancer.

**Table 2:** Associations comparing characteristics between subjects with and without thyroid dysfunction, hypothyroidism, and thyrotoxicosis.

Category	Thyroid Dysfunction	No Thyroid Dysfunction	*p*-Value	Hypothyroidism	No Hypothyroidism	*p*-Value	Thyrotoxicosis	No Thyrotoxicosis	*p*-Value
Age (years), mean ± STD	65.6 ± 10.5	65.7 ± 9.6	0.96	67.8 ± 10.1	65.1 ± 9.8	0.089	63.5 ± 10.0	66.3 ± 9.8	0.106
Male, *n* (%)	39 (51.3)	64 (49.6)	0.814	28 (58.3)	75 (47.8)	0.200	20 (47.6)	83 (50.9)	0.703
Female, *n* (%)	37 (48.7)	65 (50.3)		20 (41.7)	82 (52.2)		22 (52.4)	80 (49.1)	
Black, *n* (%)	31 (40.8)	48 (37.2)	0.792	12 (25.0)	67 (42.7)	0.135	25 (59.5)	54 (33.1)	0.034
Hispanic, *n* (%)	22 (29.0)	41 (31.8)		18 (37.5)	45 (28.7)		8 (19.0)	55 (33.7)	
White, *n* (%)	17 (22.4)	25 (19.4)		13 (27.1)	29 (18.5)		7 (16.7)	35 (21.5)	
Asian, *n* (%)	1 (1.3)	1 (0.8)		1 (2.1)	1 (0.6)		0 (0)	2 (1.23)	
Other, *n* (%)	5 (6.6)	14 (10.9)		4 (8.3)	15 (9.6)		2 (4.8)	17 (10.4)	
BMI normal, *n* (%)	28 (36.8)	49 (38.0)	0.196	13 (27.1)	64 (40.8)	0.356	18 (42.9)	59 (36.2)	0.097
Underweight, *n* (%)	7 (9.2)	11 (8.5)		5 (10.4)	13 (8.3)		5 (11.9)	13 (8.0)	
Overweight, *n* (%)	31 (40.8)	38 (29.5)		20 (41.7)	49 (31.2)		16 (38.1)	53 (32.5)	
Obese, *n* (%)	10 (13.16)	31 (24.0)		10 (20.8)	31 (19.8)		3 (7.1)	38 (23.3)	
ICIs, *n* (%)	51 (67.1)	85 (66.0)	0.671	34 (70.8)	102 (65.0)	0.325	25 (59.5)	111 (68.1)	0.432
ICIs + CTX, *n* (%)	25 (32.9)	44 (34.1)		14 (29.2)	55 (35.0)		17 (40.5)	52 (31.9)	
ICIs duration (weeks), median (IQR)	21.6 (10.0–44.0)	13.1 (5.8–31.0)	0.002	23.5 (12.1–47.4)	14.0 (6.0–31.0)	0.001	21.6 (10.0–43.1)	15.0 (6.0–33.0)	0.067
Pembrolizumab, *n* (%)	36 (47.3)	65 (50.4)	0.676	20 (41.7)	81 (51.6)	0.229	22 (52.4)	79 (48.5)	0.660
No pembrolizumab, *n* (%)	40 (52.6)	64 (49.6)		28 (58.3)	76 (48.4)		20 (47.6)	84 (51.5)	
Nivolumab, *n* (%)	23 (30.3)	42 (32.6)	0.733	17 (35.4)	48 (30.6)	0.528	12 (28.6)	53 (32.5)	0.624
No nivolumab, *n* (%)	53 (69.7)	87 (67.4)		31 (64.6)	109 (69.4)		30 (71.4)	110 (67.5)	
Atezolizumab, *n* (%)	10 (13.1)	10 (7.6)	0.208	6 (12.5)	14 (8.9)	0.464	6 (14.3)	14 (8.6)	0.267
No atezolizumab, *n* (%)	66 (86.8)	119 (92.3)		42 (87.5)	143 (91.1)		36 (85.7)	149 (91.4)	
Durvalumab, *n* (%)	10 (13.1)	16 (12.4)	0.875	8 (16.7)	18 (11.5)	0.343	4 (9.5)	22 (13.5)	0.609
No durvalumab, *n* (%)	66 (86.8)	113 (87.6)		40 (83.3)	139 (88.5)		38 (90.5)	141 (86.5)	

ICIs, immune checkpoint inhibitors; CTX, chemotherapy.

**Table 3:** Multivariate Cox regressions comparing thyroid dysfunction among patient subgroups controlling for age, gender, treatment type, and treatment duration.

	Thyroid Dysfunction	*p*-Value	Hypothyroid	*p*-Value	Thyrotoxicosis	*p*-Value
	HR (95% CI)		HR (95% CI)		HR (95% CI)	
Race/ethnicity						
Black	1.0 (ref)		1.0 (ref)		1.0 (ref)	
Hispanic	1.047 (0.5993–1.8289)	0.8720	2.466 (1.1596–5.2451)	0.0191	0.360 (0.1609–0.8052)	0.0129
White	1.140 (0.6292–2.0671)	0.6651	2.182 (0.9883–4.8185)	0.0535	0.484 (0.2082–1.1236)	0.0912
Asian or other	0.709 (0.2901–1.7306)	0.4495	1.386 (0.4782–4.0152)	0.5478	0.273 (0.0632–1.1751)	0.0812
Treatment (CTX-ICIs vs. ICIs only)	0.879 (0.519–1.442)	0.6183	0.816 (0.415–1.604)	0.5552	1.098 (0.573–2.106)	0.7773
Gender (M v F)	1.056 (0.667–1.653)	0.8342	0.7704 (0.724–2.330)	0.3801	0.982 (0.532–1.811)	0.982
ICIs duration (for every 3 week increase)	1.050 (0.994–1.1036)	0.1641	1.011 (1.007–1.058)	0.0122	1.001 (0.974–1.031)	0.8802
Age	1.005 (0.983–1.029)	0.6509	1.032 (1.000–1.065)	0.0476	0.975 (0.946–1.004)	0.0939

CTX-ICIs, concurrent chemotherapy and immune checkpoint inhibitors; ICIs, immune checkpoint inhibitors.

**Table 4:** Studies investigating the relationship of thyroid irAEs with survival outcomes.

Reference	Cancer Type(s)	Agent(s)	No. Participants	Key Results
Ferreira 2020 [48]	Melanoma, NSCLC, head and neck, Hodgkin’s lymphoma, urothelial	Pembrolizumab, nivolumab, ipilimumab	161	OS 3.27 vs. 1.76 years (*p* = 0.030) for subjects with and without thyroid dysfunction, respectively
Basak 2020 [49]	NSCLC, RCC, metastatic melanoma	Pembrolizumab, nivolumab	168	OS HR 0.18, *p* = 0.020 for subjects with thyroid dysfunction compared to those without; PFS5 HR6 0.39, *p* = 0.005 for subjects with thyroid dysfunction compared to those without
Kotwal 2020 [50]	Lung, uroepithelial, Merkel, prostate, penile	Atezolizumab, nivolumab, avelumab	91	Mortality 43.5% vs. 79.4% for subjects with and without thyroid dysfunction, respectively; median OS not reached vs. 9.8 (*p* = 0.027) for subjects with and without thyroid dysfunction, respectively
Sakakida 2019 [51]	Metastatic or unresectable malignancies	Pembrolizumab, nivolumab	150	Median OS 156 weeks vs. 59 weeks (HR 0.32, *p* = 0.001) for subjects with and without thyroid dysfunction, respectively; median PFS 66 weeks vs. 27 (HR 0.50, *p* = 0.02) for subjects with and without thyroid dysfunction, respectively
Grangeon 2019 [52]	Advanced NSCLC	Anti-PD-1 or anti-PD-L1	270	OS not reached vs. 18.2 months (HR 0.46, *p* = 0.01) for subjects with and without thyroid dysfunction, respectively; PFS 8.05 vs. 2.59 months (HR 0.56, *p* = 0.005) for subjects with and without thyroid dysfunction, respectively
Peiro 2019 [27]	NSCLC, melanoma, Hodgkin’s lymphoma	Nivolumab	73	OS HR 0.4, *p* = 0.035 in NSCLC patients, specifically with thyroid dysfunction compared to those without
Kim 2017 [26]	Stage IV NSCLC	Pembrolizumab	58	OS HR 0.11, *p* = 0.041 for subjects with thyroid dysfunction compared to those without it; PFS HR 0.38, *p* = 0.018 for subjects with thyroid dysfunction compared to those without

irAE, immune-related adverse events, NSCLC, non-small cell lung cancer; RCC, renal cell carcinoma; OS, overall survival; PFS, progression-free survival; HR, hazard ratio.

## Data Availability

The supporting data are not publicly available due to research participant privacy restrictions.
